# Apical Sealing and Bioactivity of an Experimental Gutta-Percha Containing Niobium Phosphate Bioglass

**DOI:** 10.3390/polym15071679

**Published:** 2023-03-28

**Authors:** Ruan Ferreira Sampaio, Ceci Nunes Carvalho, Vivian Bradaschia-Correa, Bruna Laís Lins Gonçalves, Victor Arana-Chavez, Alexandre P. Lima de Carvalho, Amanda Palmeira Arruda Nogueira, Renata Grazziotin-Soares, José Bauer, Giulio Gavini, Edilausson Moreno Carvalho

**Affiliations:** 1 Dentistry Postgraduate Program, University Ceuma, R. Josué Montello, 1, Renascença II, São Luís 65075-120, Brazil; 2 Pathology Core, The Centre for Phenogenomics, 25 Orde St, Toronto, ON M5T 3H7, Canada; 3 Department of Biomaterials and Oral Biology, School of Dentistry, University of São Paulo (FOUSP), Av. Prof Lineu Prestes, 2227, São Paulo 05508-000, Brazil; 4 Department of Restorative Dentistry, School of Dentistry, University of São Paulo (FOUSP), Av. Prof Lineu Prestes, 2227, São Paulo 05508-000, Brazil; 5 Dentistry Biomaterials Laboratory (Biomma), School of Dentistry, Federal University of Maranhão (UFMA), Av. dos Portugueses, 1966, São Luís 65080-805, Brazil; 6 Endodontics Division, Department of Oral Biological and Medical Sciences, Faculty of Dentistry, University of British Columbia (UBC), Vancouver, BC V6T 1Z4, Canada

**Keywords:** root canal obturation, dental infiltration, biological processes

## Abstract

This study evaluated the apical sealing ability and bioactivity of an experimental gutta-percha containing niobium phosphate bioglass. Thirty-six human premolars were endodontically prepared and divided into three groups: GPC—filling with conventional gutta-percha; GBC—filling with bioceramic gutta-percha (EndoSequence BC); GNB—filling with experimental gutta-percha containing niobophosphate. Teeth were stored in tubes containing 2 mL of simulated body fluid (SBF) solution in an oven for 30 days. Then, the samples were immersed in lanthanum nitrate solution and analyzed for apical nanoleakage (NI) with a scanning electron microscope (SEM/EDS) and transmission electron microscope (TEM). Gutta-percha specimens were immersed for 28 days (SBF) and analyzed in SEM/EDS and X-ray diffraction (XRD) to assess bioactivity. NI data originated from the SEM/EDS were analyzed using the Kruskal–Wallis test (α = 5%). NI data originated from TEM and bioactivity were descriptively reported. Statistical analysis did not detect a significant difference between groups (*p* = 0.13) for NI. In the bioactivity analysis, an abundant layer of hydroxyapatite was identified only in the surface of the GNB group samples. The gutta-percha containing niobophosphate bioglass promoted an apical sealing similar to EndoSequence BC, in addition to demonstrating bioactivity through the deposition of hydroxyapatite on the surface of the material after immersion in SBF.

## 1. Introduction

The sealing of the root canal system with biocompatible and dimensionally stable filling materials plays a fundamental role in the success of endodontic treatment, ensuring that there are no voids that can be filled later by microorganisms [1]. Thus, gutta-percha cones and endodontic cements are considered essential materials in the final phase of endodontic therapy, allowing the filling of the root canal system [2]. This sealing can be achieved according to the technique and the filling material used, which must have adequate physical-chemical and biological properties, generally related to factors such as flow capacity, viscosity, and good adhesion to the substrate [3]. Therefore, failure in any of these processes can lead to a compromised sealing, allowing contamination by fluids and microorganisms present in the oral cavity [2].

Most commercially available products are considered bioinert as they do not stimulate an active biological response when in contact with living tissues [4]. However, in recent decades, the use of bioactive glasses has been highlighted by the ability of these materials to stimulate the formation of a mineral layer when in contact with body fluids [5]. Applications of these particles have been demonstrated in several areas of dentistry such as the development of restorative materials [5,6,7] and 3D printing [8]. Recently, the use of bioactive endodontic materials has been gaining attention due to their biocompatibility and ability to stimulate the repair of dental hard tissues [9], being indicated in several situations such as the treatment of weakened teeth and regenerative endodontic therapy [10].

In this regard, the development of new filling materials containing bioactive particles capable of releasing ions, forming precipitates at the interface, and improving sealing were evaluated in the literature [11,12,13,14,15]. These particles, bioactive glasses, stand out. They are characterized by their ability to interact with body tissues/fluids and induce ion release [16], the formation of calcium and phosphate-based precipitates (bioactivity) [17], hard tissue remineralization [7], in addition to showing antibacterial activity [18]. Different compositions of these materials have been reported [19],such as niobium-based bioactive phosphate glasses [20,21]. These bioglasses are versatile and can be incorporated into different dental materials including gutta-percha [20,22].

Different in vitro methodologies have been applied to the analysis of the apical sealing capacity of filling materials with different degrees of success such as fluid filtration models and electron microscopy analyses [23,24], associated or not with the use of tracers [25]. Silver nitrate is one of the most used tracers in dentistry. Despite its wide use in micro and nanoleakage analyses, some limitations are related to the use of this substance in endodontics, often linked to factors such as the complex anatomy of the root canal system and different fluid diffusion pathways, which can generate significant variations in the results of these analyses [26,27]. Alternative elements have been used as tracers in infiltration analysis such as lanthanum. This metal is an electron-dense trivalent cation that has the ability to bind to calcium binding sites, being used as an intracellular marker and delineator of extracellular spaces [28]. Furthermore, it can be used in the analysis of oral tissues [29] and the permeability of cellular barriers [30]. Although this element can be used in ultrastructural analysis (in large magnifications), generating more reliable results, there are no studies that demonstrate the use of lanthanum solutions as a tracer in dental nanoleakage tests.

Although the use of experimental gutta-perchas containing niobium-based glasses has shown promising results in the analyses of the biocompatibility and mechanical properties [20,22], insufficient evidence about the apical sealing ability of these bioactive materials is available in the literature, especially with the use of alternative tracers such as lanthanum. Furthermore, although the bioactive potential of this material has already been suggested [20], so far there is no evidence on the mineral deposition capacity (bioactivity) of these experimental composites.

The objective of this study was to investigate the apical sealing capacity through lanthanum nanoleakage at the dentin/filling interface and the bioactivity of an experimental gutta-percha based on niobium phosphate bioglass when compared to conventional gutta-percha and bioceramic commercial gutta-percha.

## 2. Materials and Methods

### 2.1. Synthesis and Preparation of the Composite Based on Gutta-Percha and Niobium Phosphate Bioactive Glass (GNB)

**Figure 1:**
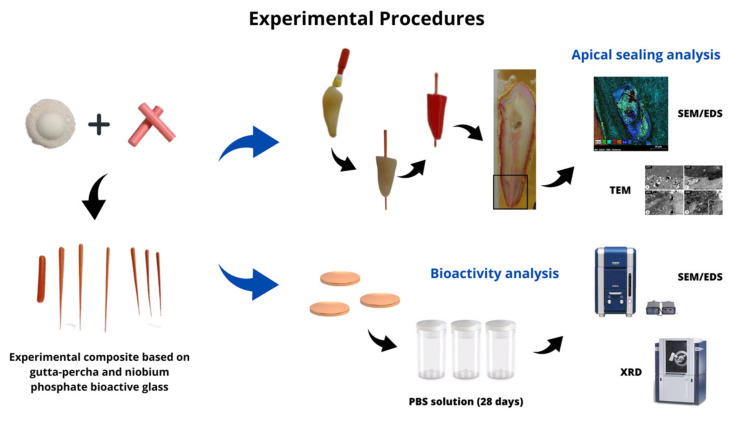
Summary of the sample preparation and experimental procedures adopted for the apical sealing and bioactivity analyses.

An experimental niobium phosphate bioactive glass was used in this study. The detailed composition and synthesis of this material have been described in previous studies [6,20]. The gutta-percha powder was placed in a mixing chamber for composite with niobium phosphate at a concentration of 30% (by weight) of the glass to the polymer, and the resulting material was inserted into an extruder. The final mixture was rolled by hand to give it a convenient format for use in endodontic fillings (0.4 cones, 0.6 accessories, and stick). The gutta-percha cones were made in an industrial production process (Odous de Deus Company, Belo Horizonte, MG, Brazil), following the quality control standards. The sample preparation and experimental procedures are summarized in Figure 1.

### 2.2. Preparation of Simulated Body Fluid (SBF)

The SBF was prepared in accordance with the standards recommended by ISO 23317:2012 [31]. During the entire SBF preparation process, the solution remained colorless, transparent, and without any deposit on the surface of the container. To prepare 1 L of SBF, 700 mL of distilled and deionized water was placed inside a plastic beaker, which was heated and magnetically stirred at 36.5 °C ± 1.5 °C. The reagents were added and dissolved one by one in the solution, so that each reagent was added to the solution after the complete dissolution of the previous reagent, respecting the following order and amounts: sodium chloride—NaCl (Sigma-Aldrich Brasil, Barueri, Brazil, ACS reagent, 8.035 g), sodium bicarbonate—NaHCO_3_ (Sigma-Aldrich Brasil, Barueri, Brazil, ACS reagent, 0.355 g), potassium chloride—KCl (Sigma-Aldrich Brasil, Barueri, Brazil, ACS reagent, 0.225 g), hydrogen dipotassium phosphate trihydrate—HK_2_O_4_P·3H_2_O (Sigma-Aldrich Brasil, Barueri, Brazil, CAS no. 16788-57-1, 0.231 g), magnesium chloride—MgCl_2_·6H_2_O (Sigma-Aldrich Brasil, Barueri, Brazil, CAS no. 7786-30-3, 0.311 g), Hydrochloric Acid—c(HCl) = 1 mol/L (Sigma-Aldrich Brasil, Barueri, Brazil, CAS no. 7647-01-0, 39 g), calcium chloride—CaCl_2_ (Sigma-Aldrich Brasil, Barueri, Brazil, CAS no. 10043-52-4, 0.292 g), sodium sulfate—Na_2_SO_4_ (Sigma-Aldrich Brasil, Barueri, Brazil, ACS reagent, 0.072 g), tris(hydroxymethyl)aminomethane—NH_2_C(CH_2_OH)_3_ (Sigma-Aldrich Brasil, Barueri, Brazil, ACS reagent, 118 g), hydrochloric acid—c(HCl) = 1 mol/L (0 to 5 g).

With a constant temperature solution, the TRIS was added incrementally until the pH reached 7.3 (QM-A338, Quimis, São Paulo, Brazil), and the temperature stabilized between 36 °C and 37 °C. Thereafter, more TRIS was added until the pH reached 7.45. After reaching these values, a 1 M HCl solution was dripped until the pH reached 7.42, and so on, until the entire quantity of TRIS was dissolved, always respecting the limits of 7.42 to 7.45 for the values of the solution pH. After total dissolution of TRIS, the solution was adjusted to a temperature of 36.5 °C ± 0.2 °C and pH 7.40 ± 0.01. The SBF was stored in a plastic container with an airtight lid and kept in a refrigerator at 4 °C.

### 2.3. Preparation and Filling of Root Canals

After approval by the Research Ethics Committee of the University of São Paulo (CAAE 0123.0.017/000-11), 36 human extracted lower premolars with fully formed apices and straight roots were selected for the study. Teeth were cleaned, tooth crowns were removed using a cutting machine (Isomet 1000, Buehler, Lake Bluff, IL, USA), and root lengths were standardized to 14 mm. 

The canals were prepared by rotary instrumentation (ProTaper, Dentsply Maillefer, Ballaigues, Switzerland) up to F5 (size 50; cone 0.05). The working length was 13 mm. The canals were irrigated with 2 mL of 1% sodium hypochlorite solution (Fórmula & Ação, São Paulo, SP, Brazil) for 1 min between instruments. The smear layer was removed with 5 mL of 17% EDTA solution (Fórmula & Ação, São Paulo, SP, Brazil) for 1 min.

The diameter of the dentin constriction in the apical region was standardized. This procedure was repeated with files of progressive size up to the #50 file, resulting in an approximately 0.56 mm/diameter. Immediately, the root canals were aspirated at the cervical, middle, and apical level with suction cannulas to remove the liquid content and dried with absorbent paper cones (Dentsply Maillefer, Petrópolis, RJ, Brazil). The external root surfaces of all teeth were dried with paper filter for further waterproofing. For this purpose, size 40 gutta-percha cones (Dentsply Maillefer, Petrópolis, RJ, Brazil) previously lubricated with water-soluble gel (Johnson & Johnson, São José dos Campos, SP, Brazil) was inserted into the root canal until it exceeded the apical foramen. External waterproofing was performed with two layers of ultra-rapid-drying nail polish, starting from the periphery of the gutta-percha cone, along the entire root and coronal extension. The root canals were then irrigated with approximately 10 mL of saline solution for complete elimination of the water-soluble gel. Afterward, the teeth were stored in individual glass containers containing the same solution in an oven at 37 °C for 48 h, in order to maintain the hydration pattern.

After preparation, the samples were randomly divided into three groups (n = 9), according to the materials used in the filling procedure: GPC Group—conventional gutta-percha (ProTaper Gutta-Percha; Dentsply Maillefer, Petrópolis, Brazil) + AH Plus cement (Dentsply Maillefer, Petrópolis, Brazil); GBC—bioceramic gutta-percha (EndoSequence BC Points, Brasseler, Savannah, Georgia, USA); GNB—experimental composite based on gutta-percha and bioactive niobium phosphate glass. For the samples from the GPC group, the AH Plus cement was manipulated according to the manufacturer’s instructions and the canals were filled using the lateral compaction technique. As for the samples from the GBC and GNB groups, the root canals were previously irrigated with 1 mL of SBF, remaining moistened until the insertion of the cones. The canals were filled with a single cone without the use of cement, using the warm vertical compaction technique with System B (SybronEndo, Orange, CA, USA) and Schilder plugs (Dentsply Maillefer, OK, USA). After obturation, all samples were restored with Z350-3M resin (3M ESPE, Dental Products, St. Paul, MN, USA) and stored in individual Eppendorf tubes containing 2 mL of PBS in an oven at 37 °C for 30 days.

### 2.4. Apical Sealing Analysis by Nanoleakage

The specimens were immersed in a 50% lanthanum solution (lanthanum nitrate—CAS 10277-43-7, Sigma-Aldrich, Saint Louis, MO, USA) and buffered using 0.1 M NaOH at 37 °C for 24 h. After this period, the teeth were washed with distilled water and dried with absorbent paper. Then, the samples were embedded in epoxy resin (Epon-Thin™, Buhler Ltd., Lake Bluff, IL, USA) and longitudinally sectioned in the mesiodistal direction using a cutting machine (Isomet 1000, Buehler, Lake Bluff, IL, USA) to expose the surface of the endodontic filling material. The interfaces were conditioned with 35% phosphoric acid solution for 5 s and washed with distilled water.

The samples were fixed, dehydrated in ascending degrees of ethanol, and a final chemical drying in hexamethyldisilazane (HMDS) (Sigma-Aldrich, St. Louis, MO, USA) was performed for 10 min. Then, the samples were coated with carbon (Sputter Coater SCD 050, BAL-TEC AG, Balzers, Liechtenstein) and the apical 5 mm of the root canal filling was divided into 5 regions of 1 mm to evaluate the nanoleakage. Samples were analyzed with a scanning electron microscope (SEM) (LEO Stereoscan 440, LEO Electron Microscopy, Cambridge, England) using the backscattered electron (BSE) mode.

To identify the lanthanum infiltrated in the apical region, energy scatter X-ray spectroscopy (EDS) analyses (INCA, Oxford, UK) were performed along the interface. Readings at lower magnification within a predetermined area (300 mm^2^) and at higher magnifications were performed to identify and determine the exact location of the lanthanum particles. Each 1 mm region was classified according to the following scores: 0 (absence of infiltration at both interfaces) and 1 (presence of infiltration in at least one of the interfaces).

### 2.5. Transmission Electron Microscopy (TEM)

For ultramorphological analysis, nine teeth (n = 3) were prepared in the same way described in Section 2.3. Samples were fixed in 2.5% glutaraldehyde containing lanthanum nitrate in sodium cacodylate buffer (0.1 M, pH 7.7) at room temperature for 1 week. After washing for 10 min in 0.1 M sodium cacodylate buffer, the specimens were postfixed in 1% osmium tetroxide containing lanthanum nitrate in sodium cacodylate (0.1 M, pH 7.7) for 3 h. After washing for 1 h in the same buffer, they were postfixed again in buffered 1% osmium tetroxide for 2 h.

All samples were dehydrated in graded concentrations of ethanol and embedded in Spurr resin (Low-Viscosity Embedding Kit, Electron Microscopic Sciences, St. Louis, MO, USA). The 2 µm thick sections stained with toluidine blue were examined with an optical microscope and the regions containing the 1.5 mm apical root canal were trimmed for ultrafine sectioning. Ultrathin sections were stained with lead citrate/uranyl acetate and collected on copper grids. Next, the samples were examined with a transmission electron microscope (TEM) (JEM 1010, JEOL, Tokyo, Japan) operated at 80 kV. Images were obtained digitally with a GATAN imaging platform equipped with a CCD camera (Orius SC1000, GATAN, Pleasaton, CA, USA) [32].

### 2.6. Bioactivity Analysis

Three disc-shaped specimens (5 mm-diameter and 1 mm-thickness) were made for each group. Gutta-percha was heated under indirect heat on a metal plate and metal matrices were filled with the material. After cooling, the samples were removed from the molds, placed in individual plastic bottles containing 5 mL of PBS, and stored in an oven at 37 °C for 28 days. After the immersion period, the specimens were removed from the solution and dehydrated in a desiccator for 24 h.

Scanning electron microscopy (SEM) images (TM3030, Hitachi, Tokyo, Japan) at 1000× and 5000× magnifications were obtained before and after immersion in SBF for morphological characterization of the precipitates deposited on the surface. Next, energy dispersive spectroscopy (EDS) spectra (Quantax, Bruker, Massachusetts, USA) were collected similarly to the SEM images. Additionally, X-ray diffraction (XRD) analyses were performed to characterize the precipitates deposited on the surface of the samples in a diffractometer (D8 Advance, Bruker, Germany) with Cu-Kα radiation (40 kV, 40 mA), linear detector, and 0.6 mm gap. All measurements were made at 25 °C, with an angular step (2θ) of 0.02° and a measurement range of 7 to 80°. To determine the phases present, the spectra were analyzed with the aid of reference diffraction data from the International Center for Diffraction Data (ICDD).

### 2.7. Statistical Analysis

Statistical analysis was performed using SigmaPlot 13 software (SigmaPlot 13.0, Systat Software Inc., San Jose, CA, USA). Apical sealing data (nanoleakage) were analyzed using the Kruskal–Wallis test with a significance level of 5%. The results of the analyses in MET and bioactivity (SEM, EDS, and XRD) were descriptively reported.

## 3. Results

### 3.1. Lanthanum Nanoleakage

**Figure 2:**
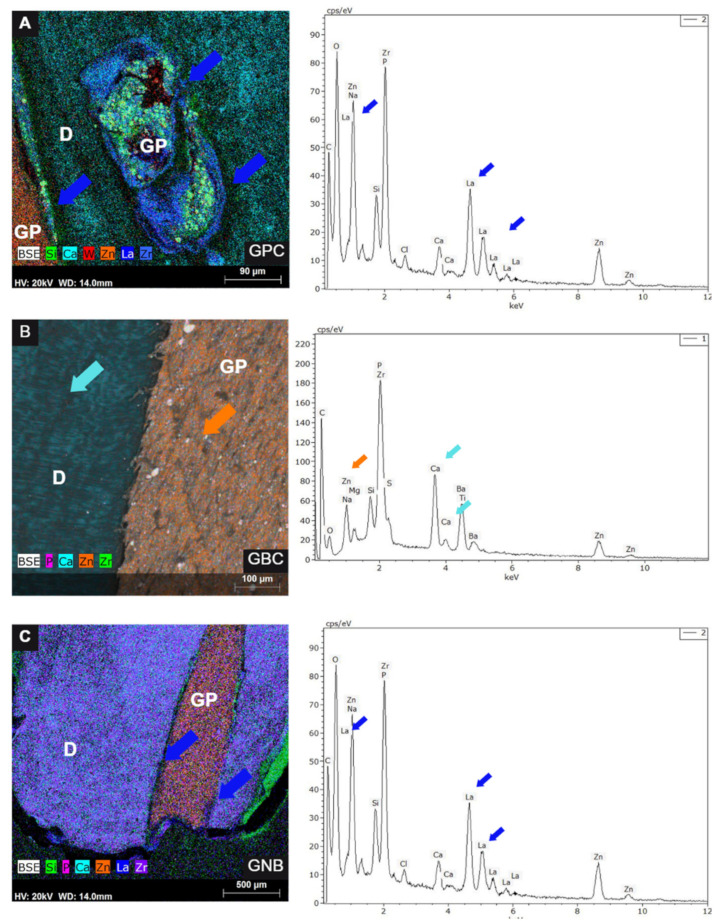
Representative images of lanthanum nanoleakage in all of the evaluated groups. (**A**) Conventional gutta-percha (GPC) group. The presence of lanthanum at the interface is indicated by blue arrows. (**B**) EndoSequence BC Sealer (GBC) group. The EDS analysis showed the presence of elements such as calcium (blue arrow) and zinc. (**C**) Gutta-percha group with niobium phosphate glass (GNB). The presence of lanthanum at the interface is indicated by blue arrows. Markers: D—dentin; GP—gutta-percha.

The results of the lanthanum nanoleakage analysis are shown in Table 1 and Figure 2. The SEM/EDS images demonstrated the presence of lanthanum in samples from the GPC (Figure 2A) and GNB groups (Figure 2C). No sample from the GBC group showed lanthanum at the interfaces (Figure 2B). The Kruskal–Wallis test did not detect a statistically significant difference between groups (*p* = 0.13).

### 3.2. TEM Analysis

**Figure 3:**
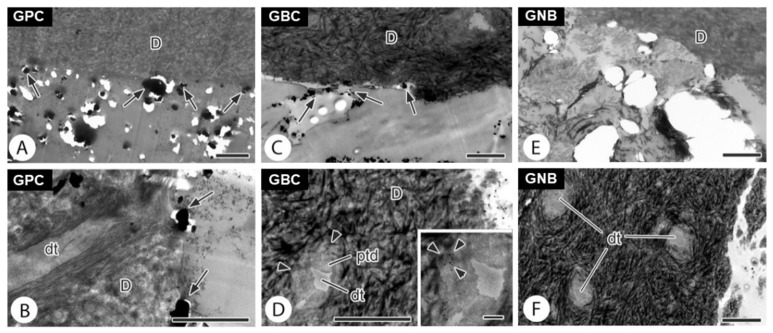
Representative transmission electron microscopy (TEM) images of the GPC (**A**,**B**), GBC (**C**,**D**), and GNB (**E**,**F**) groups. Presence of lanthanum at the interface (black arrows) in the GPC and GBC groups. DT: tubular dentin, D: dentin, PTD: peritubular dentin. (Scale bar: 800 nm; figure D zoom—200 nm).

The ultrastructural analysis of the dentin/filling material interface did not detect lanthanum nitrate particles infiltrated in specimens filled with GNB (Figure 3E,F). In contrast, the root canals filled with GPC (Figure 3A,B) and GBC gutta-percha (Figure 3C,D) showed numerous lanthanum nitrate particles infiltrated at the interface between the root dentin and filling materials. Lanthanum nitrate particles were detected within the dentinal tubules in roots filled with GBC (Figure 3D).

### 3.3. Bioactivity

**Figure 4:**
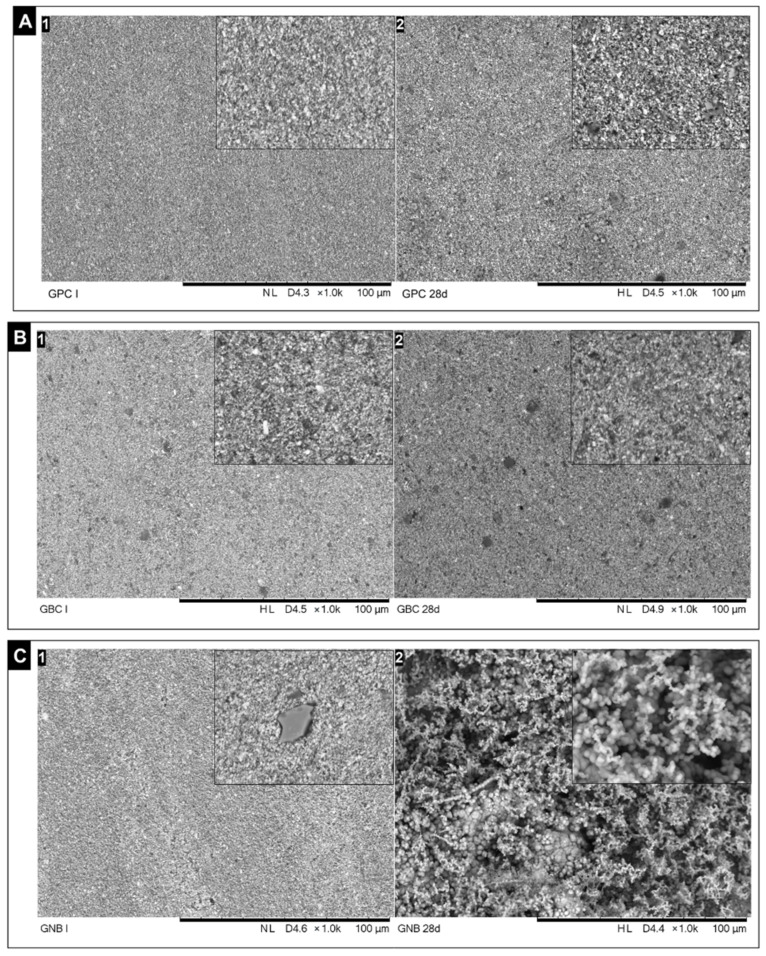
Representative SEM images before (1) and 28 days (2) after immersion in SBF of all evaluated groups. (Magnification: ×1000, scale: 100 μm). (**A**) GPC group. Absence of precipitate formation on the surface of the material. (**B**) GBC group. Absence of precipitate formation on the surface of the material. (**C**) GNB group. 1—in the initial images, glass particles containing niobium on the surface of the material were present. 2—after 28 days immersed in SBF, a large volume of precipitates indicative of bioactivity on the surface was noted.

Representative images in the SEM of the bioactivity analyses before and after immersion in PBS are shown in Figure 4. In the GPC and GBC groups, it was not possible to observe the formation of precipitates after immersion (Figure 4A,B). As for the gutta-percha group containing glass niobium phosphate (GNB), it was possible to observe a large formation of spherically shaped precipitates, indicative of the deposition of bioactive precursors on the surface of the material (Figure 4C).

**Figure 5:**
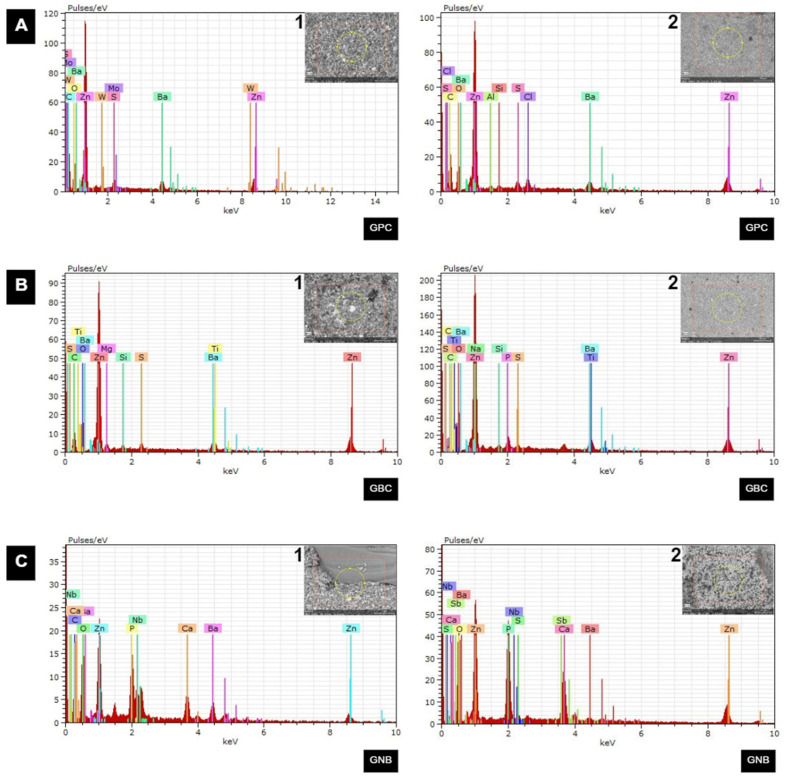
Representative EDS analysis images of the samples in all of the evaluated groups. (**A**) GPC group. Presence of elements such as Ba (barium) and Zn (zinc), W (tungsten), S (sulfur), MO (molybdenum), C (carbon), and O (oxygen) before (1) immersion in SBF. Presence of Zn, Ba, S, C, SI (silicon), and AL (aluminum) appeared after 28-days of immersion (2) in SBF. (**B**) GBC group. Presence of elements such as Ba, Zn, NB (niobium), P (phosphorus), O, CA (calcium), C, and S before (1) immersion in SBF. Presence of Ba, Zn, P, O, C, S, NA (sodium), and TI (titanium) appeared after 28 days of immersion (2) in SBF. (**C**) GNB group. Presence of elements such as Nb, Ca, P, C, Ba, Zn, and O before (1) immersion in SBF. Presence of Nb, Ca, P, C, Ba, Zn, O, SB (antimony), and S appeared after 28 days (2) of immersion in SBF, and there was a substantial increase in the intensity of the Ca and P peaks, attributed to the formation of precipitates on the surface of the material.

Representative images of the EDS analysis before and 28 days after immersion in SBF are shown in Figure 5. For the GPC and GBC groups, surface spectroscopy of the samples before immersion showed the presence of peaks of elements such as barium, zinc, silicon, tungsten, titanium, and aluminum. The same pattern was observed after 28 days of immersion, confirming that there was no formation of precipitates (Figure 5A,B) on the surface of these materials. On the other hand, for the samples from the GNB group (Figure 5C), the initial EDS readings confirmed the presence of niobium phosphate glass particles on the surface of the samples, indicated by the presence of niobium, calcium, and phosphorus, in addition to barium and zinc. After 28 days of immersion, a substantial increase in the intensity of the calcium and phosphorus peaks was noted, which can be attributed to the precipitates that formed on the surface of the material containing niobium phosphate glass.

**Figure 6:**
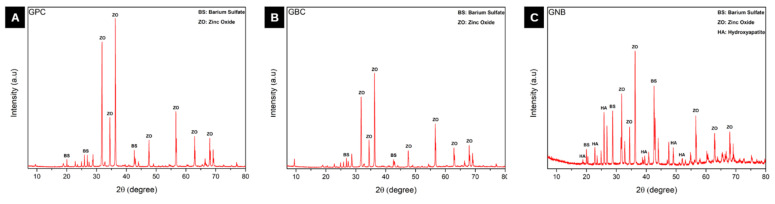
Representative spectrum of the XRD analysis of samples from all evaluated groups. (**A**) GPC group after 28 days of immersion in SBF. Presence of patterns corresponding to barium sulfate and zinc oxide; (**B**) GBC group after 28 days of immersion in SBF. Similar to the GPC group with the presence of patterns corresponding to barium sulfate and zinc oxide. (**C**) GNB group after 28 days of immersion in SBF. In addition to the presence of patterns corresponding to barium sulfate and zinc oxide, there was the presence of hydroxyapatite, attributed to the formation of precipitates identified in SEM/EDS.

X-ray diffraction spectra of the gutta-percha samples evaluated after 28 days of immersion in SBF are shown in Figure 6. For the samples from the GPC group, peak patterns corresponded to barium sulfate (ICDD card #01-080-0512) and zinc oxide (ICDD card #01-074-0534) (Figure 6A). Similarly, in the samples of the GBC group, the same compounds were observed (barium sulfate—ICDD card #01-080-0512; zinc oxide—ICDD card #01-079-0206) (Figure 6B). In the samples of the GNB group, in addition to the presence of compounds identified in the other groups evaluated (barium sulfate—ICDD card #00-024-1035; zinc oxide—ICDD card #01-079-0206), it was possible to confirm the presence of peak patterns corresponding to hydroxyapatite (ICDD card #01-073-0293), confirming the deposition of bioactive precursors on the surface of the gutta-percha samples containing glass niobium phosphate (Figure 6C).

## 4. Discussion

The addition of bioactive glasses in dental materials favors the release of ions that can stimulate mineral deposition at the interfaces and contribute to the remineralization of dental hard tissues [5]. The results of the present study showed that the experimental composite containing bioactive niobium phosphate glass had an apical sealing capacity similar to the commercial groups, with the advantage of showing substantial bioactivity verified through the deposition of a superficial layer of hydroxyapatite, which was not observed for the other evaluated groups.

Gutta-percha is well-tolerated by living tissues and does not negatively interfere with the periapical healing process following root canal treatment. However, one of the biggest problems related to the failure of these treatments is the lack of apical sealing [33,34]. Although there is no consensus on the methods used to evaluate apical sealing in endodontics, one of the main in vitro models used is the analysis of micro and nanoleakage with tracer substances such as silver nitrate [27]. Silver nitrate presents nanometric particles with molecules of high molecular weight, presenting a good penetration capacity with superior results to organic dyes such as methylene blue [35]. Nevertheless, in contact with the dental substrate, silver nitrate can deposit silver phosphate crystals inside dentinal tubules, which would limit the penetrating power of this tracer [36]. In addition, other factors such as pH and silver concentration in the solution can influence nanoleakage results such as the occurrence of false positives [37].

On the other hand, lanthanum has the ability to penetrate very small spaces, being an interesting alternative for ultrastructural analysis such as those performed in TEM [38]. This element does not have the ability to cross cells unless the membrane is damaged, being considered a very efficient extracellular marker [39]. In dental tissues such as enamel, lanthanum acts as a probe for calcium binding sites [38], showing a high affinity for this element [40], and can be considered as a suitable tracer for the nanoleakage of dental tissues.

In the present study, the analysis of apical sealing by nanoleakage with lanthanum nitrate in SEM/EDS demonstrated its presence in samples from the GPC (Figure 2A) and GNB groups (Figure 2C), while none of the samples from the GBC group showed lanthanum at the interfaces (Figure 2B). Despite these results, the ability of gutta-percha with bioactive particles filled without the use of endodontic sealer was considered to be similar to the control group, in which the canals were filled by lateral compaction with AH Plus.

In the GPC group, only ~10% of the samples had leakage. These results are in line with the previous literature [41,42,43] and are attributed to the adhesion ability of AH Plus to the root canal walls and its good physical-chemical properties [44,45]. However, AH Plus does not have the high biocompatibility and bioactive potential as other sealers, for instance, bioceramics [46].

On the other hand, the sealing results found for the GBC and GNB groups may highlight the positive role that bioactive particles have at the dentin/filling material interface. For these groups, a thermoplastic/filling technique without the use of cement in a humid environment (with SBF solution) was adopted, which favored the interaction between the bioactive particles and dentin walls. This direct interaction stimulated mineral deposition at the interface (bioactivity) that contributed to the sealing observed in these groups. In a previous study, the composite based on experimental gutta-percha and niobium phosphate glass, filled under the same conditions, presented similar bond strength results to the control group (gutta-percha + AH Plus) [20].

In the apical seal analysis using TEM, there were no infiltrated samples in the region of the dentinal tubules of the GNB group, although infiltrated samples were found in the SEM/EDS analysis, reinforcing the importance of bioglasses for a better sealing at the ultrastructural level. The ability of bioactive particles to release ions and deposit minerals may have been responsible for the obturation of the dentinal tubules, ensuring the sealing observed in the samples of this group.

Studies have shown that the inclusion of niobium in phosphate glasses confers greater chemical stability, better mechanical properties [47,48,49], and induces the formation of bioactive precipitates similar to silicate glasses [50,51]. In the present study, experimental bioactive gutta-percha (GNB) showed promising bioactivity results, demonstrating that the incorporation of niobium glass was able to make gutta percha bioactive. After 28 days of immersion in PBS, samples from these groups exhibited the formation of spherical precipitates, indicative of bioactivity (Figure 4C). In contrast, for the GPC and GBC groups, there was an absence of precipitates and/or peaks of bioactive precursors (Figure 4A,B). Characterization of the precipitates via EDS and XRD confirmed the deposition of an abundant layer of hydroxyapatite, rich in calcium and phosphorus, on the surface of all samples from the GNB group (Figure 6C).

The application of SBF in the root canal at the time of filling simulated the real conditions of dentin moisture. Although some studies have already shown that an excessive humid environment can negatively influence the mechanical properties of conventional filling materials [52,53,54], this condition had a positive effect on the sealing of samples from the GBC and GNB groups, probably related to the presence of bioactive particles in these materials. Previous evidence [55] demonstrated that the rewetting of root dentin with SBF improved the bond strength of bioactive filling materials.

As in GNB, gutta-percha Endosequence BC had bioactive particles (bioceramics). However, the results of this group did not demonstrate the same bioactivity capacity of the GNB group after 28 days of immersion in SBF. These findings may be related to the composition and development of these materials: in the experimental gutta-percha bioglasses, particles were present throughout its interior while in the bioceramic gutta-percha, the bioactive particles were only present on the surface [20]. In this context, the method used in this study to prepare the bioactive samples (heating and filling a metallic matrix) may have isolated the bioactive particles in the gutta-percha matrix, preventing the interaction with the SBF. In addition, in the experimental gutta-percha, 30% (by weight) of particles were incorporated, a value higher than that found on the surface of the gutta-percha Endosequence BC.

Experimental gutta-percha containing bioactive niobium phosphate glass proved to be a promising material, with satisfactory results in apical sealing and bioactivity compared to the tested commercial materials. The sealability (i.e., the potential to achieve a three-dimensional seal of the root canal system) is favored by the material bioactive potential and prevents apical percolation of fluids and root canal reinfection.

## 5. Conclusions

The experimental gutta-percha containing bioactive niobium phosphate glass promoted apical sealing, which was similar to the commercial-gutta-percha and the bioceramic-gutta-percha, and had bioactivity, ass confirmed by the deposition of a layer of hydroxyapatite on its surface after immersion in SBF. The addition of bioactive niobium phosphate glass into gutta-percha is advantageous in relation to the traditional gutta-percha only in relation to the bioactive potential.

## Figures and Tables

**Table 1:** Distribution of the presence of nanoleakage by lanthanum in the GPC (conventional gutta percha), GBC (BC Sealer gutta percha), and GNB (niobium phosphate gutta percha) groups.

Groups	Nanoleakage		*p*-Value
	Absent	Present	
GPC	8	1	*p* = 0.13 *
GBC	9	0	
GNB	6	3	

* Kruskal–Wallis test (α = 0.05).

## Data Availability

All of the research data used in this manuscript will made be available upon requested.
